# Speech and swallow rehabilitation in head and neck cancer: United Kingdom National Multidisciplinary Guidelines

**DOI:** 10.1017/S0022215116000608

**Published:** 2016-05

**Authors:** P Clarke, K Radford, M Coffey, M Stewart

**Affiliations:** 1 Department of ENT, Charing Cross and Royal Marsden Hospitals, London, UK; 2 Sandwell and West Birmingham NHS Trust, Birmingham, UK; 3 Department of Speech and Language Therapy, Head and Neck/Oncology, Charing Cross Hospital, London, UK; 4 ENT Department, Royal Alexandra Hospital, Paisley, UK

## Abstract

This is the official guideline endorsed by the specialty associations involved in the care of head and neck cancer patients in the UK. The disease itself and the treatment can have far reaching effects on speech and swallow function, which are consistently prioritised by survivors as an area of concern. This paper provides recommendations on the assessments and interventions for speech and swallow rehabilitation in this patient group.

**Recommendations:**

• All multidisciplinary teams should have rehabilitation patient pathways covering all stages of the patient's journey including multidisciplinary and pre-treatment clinics. (G)

• Clinicians treating head and neck cancer patients should consult the National Cancer Rehabilitation Pathway for head and neck cancers. (G)

• All head and neck cancer patients should have a pre-treatment assessment of speech and swallowing. (G)

• A programme of prophylactic exercises and the teaching of swallowing manoeuvres can reduce impairments, maintain function and enable a speedier recovery. (R)

• Continued speech and language therapist input is important in maintaining voice and safe and effective swallow function following head and neck cancer treatment. (R)

• Disease recurrence must be ruled out in the management of stricture and/or stenosis. (R)

• Continuous radial expansion balloons offer a safe, effective dilation method with advantages over gum elastic bougies. (R)

• Site, length and completeness of strictures as well as whether they are in the presence of the larynx or not, need to be assessed when establishing the likelihood of surgically improved outcome. (G)

• Primary surgical voice restoration should be offered to all patients undergoing laryngectomy. (R)

• Attention to surgical detail and long-term speech and language therapist input is required to optimise speech and swallowing after laryngectomy. (G)

• Patients should commence wearing heat and moisture exchange devices as soon as possible after laryngectomy. (R)

## Introduction

Most head and neck cancers and their treatments affect speech and swallowing and this section therefore concentrates on the rehabilitation of these functions.^1^^–^^6^ Allied health professional (AHP) head and neck cancer rehabilitation pathways are required as part of the implementation of the Improving Outlines Guidance rehabilitation measures and are required for peer review. These pathways should cover all stages of the patient's journey from diagnosis, through treatment, to survivorship and end of life care and should include relevant intervention from dietetics, physiotherapy, occupational therapy and speech and language therapy. Pathways for oral rehabilitation with input from hygienists, restorative dentists, dental implantologists, prosthetic technicians should also be considered.

The stages of the pathways and the allied health professional interventions appropriate to each stage are detailed along with an extensive evidence review in the National Cancer Rehabilitation Pathway for Head and Neck Cancers.^2^

Responsibility for the rehabilitation of voice, speech and swallowing rests with the whole multidisciplinary team (MDT), but is the specific role of the speech and language therapist within this team. Speech and language therapists should discuss their role and outline the need for the patient's active participation in therapy to maximise outcomes. The patient's family and carers are also involved in this rehabilitation. Within the MDT, the decision on an appropriate course of treatment should take into account the effects on functions such as voice, speech and swallowing as well as survival so as to suit each individual's preferences and lifestyle.

**Recommendations**

• All MDTs should have rehabilitation patient pathways covering all stages of the patient's journey including multidisciplinary and pre-treatment clinics (G)
• Clinicians treating head and neck cancer patients should consult the National Cancer Rehabilitation Pathway for Head and Neck Cancers (G)

## Rehabilitation of voice, speech and swallow

### Goals of rehabilitation

• Achieve the best possible functional outcome and quality of life (QoL)
• Identify and carry out interventions which are most effective for both the specific treatment and the individual patient at the optimal time
• Provide support and rehabilitation to patients and their carers.

### Assessment

All head and neck cancer patients should have a pre-treatment assessment of speech and swallowing.^1^^–^^6^ Baseline assessments should be undertaken by the speech and language therapist and appropriate interventions to maintain functions before treatment should be undertaken. Assessments of voice, speech and swallowing should be carried out at all stages of the pathway.

Clinical assessments include: oral-motor examination (lip closure, range of motion), articulation, tongue control and strength; evaluation of the oropharyngeal swallow (timing, efficiency, aspiration, tongue and laryngeal motion) and perceptual evaluation of voice quality.

Instrumental assessments of swallowing include flexible endoscopic examination of swallowing, videofluoroscopy and/or modified barium swallow.^5^ Instrumental assessments of voice include: endoscopy, stroboscopy and speech studio/laryngograph. These assessments can provide useful biofeedback to patients and demonstrate the effectiveness of interventions.

### Therapy/interventions

#### Pre-treatment

Pre-treatment counselling by AHP teams should be provided to advise on the anticipated effects of the cancer as well as subsequent treatments (chemoradiation, radiotherapy (RT), surgery and palliation).^7^

A strict programme of prophylactic exercises and the teaching of swallowing manoeuvres can reduce specific impairments, maintain functions and enable a speedier recovery ensuring post-treatment rehabilitation is more successful.^8^ For those undergoing surgery the teaching of swallow strategies beforehand can reduce risk and maximise function. This may also reduce the need for tube feeding during treatment and the length of post-treatment tube feeding.

#### Post-treatment

##### Voice

Specific therapy techniques can be targeted at projection, pitch, reduction of fatigue, increased adduction, coordination of respiration, vocal hygiene and amplification. These are particularly relevant to those having laser surgery or RT to the larynx.

##### Speech

For those undergoing oral resections a programme of compensations, articulation and intelligibility can be started once suture lines have healed.

##### Swallowing

Following instrumental assessment, interventions should be targeted at specific physiological deficits and volitional control to compensate for the changes to the anatomy and physiology. This can reduce the risk of aspiration, malnutrition and improve QoL. These interventions include:^9^

• Postures to reduce aspiration, e.g. head turn, chin tuck
• Manoeuvres, e.g. supraglottic swallow, Mendelsohn.
• Therapeutic exercises, e.g. thermal tactile stimulation, range of motion, shaker
• Diet modifications regarding textures and recommendations on oral or non-oral intake.

#### Oral rehabilitation

Intra-oral prostheses providing palatal lift, obturation and augmentation can improve speech and swallow function after oral resections and the speech and language therapist and restorative dental surgeon and/or prosthetic technician need to work closely together. Radiation-induced fibrosis can present with trismus. This can cause pain, difficulty with oral intake, poor oral hygiene and lack of dental care. Exercises with tongue depressors or a specific device can increase mouth opening.

**Recommendations**

• All head and neck cancer patients should have a pre-treatment assessment of speech and swallowing (G)
• A programme of prophylactic exercises and the teaching of swallowing manoeuvres can reduce impairments, maintain function and enable a speedier recovery (R)
• Continued speech and language therapist input is important in maintaining voice and safe and effective swallow function following head and neck cancer treatment (R)

## Management of stenosis and stricture

### Prevention, assessment and diagnosis

Dysphonia following RT and chemoradiotherapy to the oro/hypopharynx is multifactorial and difficult to treat. Xerostomia, loss of tongue base bulk and fibrosis/reduced function of constrictors all play a part. Speech and language therapy and AHPs’ input as above remains of utmost importance, but stenosis and stricture can also develop.

Stenosis of the (hypo)pharynx and neopharynx is common following treatment for laryngeal and pharyngeal cancer.^10^^,^^11^ After treatment of cervical oesophageal cancer some degree of stenosis is almost inevitable in this region especially following CRT.^6^ Reported rates vary from 8 per cent following primary chemoradiotherapy to 40 per cent or more following salvage surgery after (chemo)radiotherapy, particularly if preceded by a pharyngocutaneous fistula.^10^ Additional dysphagia occurs in extended surgery, particularly with posterior tongue resection and with extended neck surgery with sacrifice of glossopharyngeal and hypoglossal nerves (lesser), and vagus nerve (major).^12^^,^^13^

No standardised definition exists to help to measure stenosis rates. Anatomical stenosis might be of greatest interest to the surgeon, but functional stenosis is of no less impact and interest to the patient. Videofluoroscopy, supplemented by axial imaging, is the tool best able to identify the nature of a stenosis of the (neo)pharynx and assess the degree of impact on swallowing. Importantly, barium swallows also have the capacity to identify a proportion of occult recurrences masquerading as benign stenosis.

Predictors of stenosis are helpful to surgeons. Studies have shown that following laryngectomy and partial pharyngectomy a 3 cm (unstretched) to 8 cm (stretched) posterior pharyngeal strip is sufficient to allow normal post-treatment swallow and voice rehabilitation. Circular/circumferential rather than linear scars remain more stenosis prone, but no data exist on the minimum luminal diameter with a circular scar to allow normal swallowing. Repair of the suprahyoid muscles (which include the middle constrictor) to the thyropharyngeus muscles after laryngectomy has been advocated and may improve swallow by reducing the size and effect of a pseudoepiglottis as well as allowing better function of the middle constrictor. Cricopharyngeal myotomy and horizontal closure of the pharynx with laryngectomy is generally held to improve speech and swallow outcomes especially when performed with primary tracheo-oesophageal puncture and valve reconstruction for speech rehabilitation. In addition, the relationship between luminal diameter and the use of peristaltic *vs* non-peristaltic flaps have yet to be quantified in maintaining a functional post-operative voice and swallow.

The role of salivary bypass tubes may reduce fistula rates and hence possible stricture rates, but this needs further study.

### Treatment

This depends on the type (functional *vs* anatomical, scar *vs* recurrence), site and comorbid factors such as fitness for further reconstructive surgery. Median feeding tube placement times following all forms of treatment for head and neck cancer are in the region of 20–26 weeks, and up to 50 per cent of patients reconstructed with free or pedicle flaps are tube-feed dependent at one year post-surgery. Reported rates of complication with percutaneous endoscopic gastrostomy and radiologically inserted gastrostomy tubes vary considerably with up to 3 per cent mortality rates reported in some series and 10 per cent significant complication in others. Clearly the use of different supplemental feeding techniques will depend on local experience in this respect.

Dilation of isolated short segment strictures remains a valuable means of controlling symptoms for patients with poor life expectancy or multiple comorbidities.^10^ Continuous radial expansion balloons allow dilation up to 20 mm diameter and may be safer and more effective than traditional bougies. They can also be utilised without general anaesthesia. It is clear that many patients require multiple dilations, often without long-lasting relief of dysphagia.

Sternomastoid flaps can be useful in the non-irradiated patient, but are less reliable than pectoralis major, radial forearm flap (RFF), anterolateral thigh (ALT) and jejunal flaps. Choice of and reasons for a particular free flap vary depending on familiarity with the flap and perceptions of function *vs* cosmesis. Reported case series for RFF, jejunum or ALT describe similar complication rates (<5 per cent flap failure, up to 50 per cent pharyngocutaneous fistula) and success rates (speech intelligibility and swallow performance).^14^

The length and completeness of stenosis are important factors in advising patients whether significant improvement can be obtained. Complete stricture of the hypopharynx post-chemoradiotherapy can be improved with total laryngopharyngectomy, but patients need to be warned that swallowing outcomes are often poorer in this group than primary pharyngectomy patients.

### Cricopharyngeal myotomy

Cricopharyngeal myotomy appears to have little value *per se* for improvement of dysphagia following surgical treatment of cancers of the oropharynx.^15^ In combination with vocal fold medialisation, where needed, and laryngeal elevation, better success rates may be obtained.

**Recommendations**

• Disease recurrence must be ruled out in the management of stricture and stenosis (R)
• Continuous radial expansion balloons offer a safe, effective dilation method with advantages over gum elastic bougies (R)
• Site, length and completeness of strictures as well as whether they are in the presence of the larynx or not, need to be assessed when establishing the likelihood of surgically improved outcome (G)

## Rehabilitation after laryngectomy

### Speech

Laryngectomy results in significant alteration of anatomy and often complex rehabilitation. A range of voice prostheses are now available, with Blom Singer and Provox being the commonly used ones. If visual, cognitive and fine motor skills are intact, independence should be fostered by teaching patients to self change their voice prostheses. Where appropriate, ‘hands-free’ outer valves should be available for patients to try. Although surgical voice restoration techniques dominate, it is important to consider the use of oesophageal speech and electrolarynges. Electrolarynges use an external vibratory source and are either placed in the mouth or against the neck or cheek to produce sound. Both these methods can have their place in the rehabilitation process.^16^

Speech and language therapists with appropriate training and expertise in the management of the stoma and tracheo-oesophageal puncture should be part of all MDTs. The MDT should ensure that there are procedures to manage out of hours problems such as loss or aspiration of prosthesis. Patients and local teams should be aware that if a prosthesis cannot be replaced the puncture should be kept patent with a catheter or stent for instance. Speech and language therapists should be aware of the need for and rationale behind, amongst others, videoflouroscopy for troubleshooting, botulinum toxin, antifungals, management of leakage through as well as peripheral leakage around a prosthesis. The Royal College of Speech and Language Therapists has recently published an excellent and comprehensive document covering these topics: ‘Prosthetic Surgical Voice Restoration (SVR): The role of the speech and language therapist’.^1^

### Swallow

There has been a growing appreciation in recent years that swallowing also requires rehabilitation in laryngectomy patients.^16^^–^^18^ Although laryngectomy patients should not aspirate unless their voice prosthesis is leaking, they may have difficulty swallowing solid foods or take significantly longer than others to finish meals. It has been suggested that as many as 42 per cent of laryngectomy patients have a degree of dysphagia three years post-surgery with a 72 per cent incidence of self-reported dysphagia. Higher levels of depression and anxiety have also been documented in laryngectomees who have dysphagia.^19^ Videofluoroscopy is one of a number of swallow evaluation tools used with laryngectomy patients and can contribute to surgical consideration of interventions such as botulinum toxin and dilatation to treat dysphagia. Further rehabilitation tools include the use of exercises to strengthen specific muscles such as tongue base. Appetite can also be affected by a significant loss of ability to taste and smell after laryngectomy. Olfactory rehabilitation utilising the ‘polite yawn’ has been proposed to help correct this.

### Respiration

Respiration is altered significantly post-laryngectomy with the patient now breathing through an open neck stoma bypassing the nasal passages and throat. As a consequence of this anatomical change, the ability to filtrate irritants such as dust from the air and to humidify inhaled air is lost. This can result in increased mucus production and crusting of dried secretions. In recent years, humidification exchange devices have been developed to restore humidification and filtration. Rehabilitation of pulmonary function should be offered to all laryngectomy patients and should involve education about the use of stoma covers and bibs. The presence of an open neck stoma causes some patients anxiety and rehabilitation may include such diverse subjects as advice about maintaining appearance and showering safely.

The adjustment to life as a laryngectomee can be significant. Tools such as the EORTC Core Quality of Life Questionnaire and the University of Washington Quality of Life Tool, version 4 can be useful in identifying not only those at risk of psychosocial problems but also to help plan and focus rehabilitation.^19^

**Recommendations**

• Primary surgical voice restoration should be offered to all patients undergoing laryngectomy (R)
• Attention to surgical detail and long-term speech and language therapist input is required to optimise speech and swallowing after laryngectomy (G)
• Patients should commence wearing heat and moisture exchange devices as soon as possible after laryngectomy (R)

### Key points

• Speech and swallow rehabilitation needs should be assessed before treatment
• Assessment and appropriate interventions should take place throughout the patient journey, including ongoing after treatment
• Multidisciplinary assessment and management of swallowing problems is important
• Videoflouroscopy is an important tool in assessing swallow problems
• Dysphagia caused by pharyngeal stenosis after chemoradiotherapy can be difficult to correct and complex cases should be managed by expert teams.
